# Terrorism, Perceived Threat, and Support for Surveillance: A Virtual Reality Experiment on Cyber vs. Conventional Terrorism

**DOI:** 10.3390/ijerph22111634

**Published:** 2025-10-27

**Authors:** Keren L. G. Snider, Amit Cohen, Giulia Dal Bello, Guy Baratz, Béatrice S. Hasler, Daphna Canetti

**Affiliations:** 1 Department of Politics & Communication, Jerusalem Multidisciplinary College (JMC), Jerusalem 9101001, Israel; 2 School of Political Sciences, University of Haifa, Haifa 3498838, Israel; 3 Sagol School of Neuroscience, Tel Aviv University, Tel Aviv 6997801, Israel; 4 Department of Information Systems, Business School, University of Liechtenstein, 9490 Vaduz, Liechtenstein

**Keywords:** exposure to terrorism, cyberattacks, mental health, psychological distress, threat perception, surveillance policy, critical infrastructure, virtual reality experiment, resilience

## Abstract

Governments worldwide are increasingly adopting intrusive surveillance measures to counter terrorism. However, the psychological and public health consequences of exposure to terrorism remain underexplored. This study examines how exposure to cyber and conventional terrorism affects perceived national threat and support for surveillance policies, using a controlled virtual reality experiment in which participants were immersed in realistic simulations of lethal terror attacks targeting critical railway infrastructure in Israel. Participants were randomly assigned to one of three conditions: cyber (*N* = 59), conventional (*N* = 59), or control (*N* = 45). Outcomes were standardized, but the framing differed by type of attack. Findings show that perceived national threat perceptions are a key mechanism linking exposure to terrorism to surveillance attitudes. At lower threat levels, participants differentiated between cyber and conventional attacks. In contrast, heightened threats led to uniform support for expansive surveillance regardless of the attack modality. Results demonstrate that exposure to terrorism, including cyberattacks on critical infrastructure, can activate psychological responses with implications for public resilience and policy attitudes, shaping preferences concerning privacy and security. These findings underscore the broader societal and public health relevance of understanding how people react to evolving security threats that disrupt essential systems such as transportation, energy, and healthcare.

## 1. Introduction

Terrorism, whether conventional or cyber-based, poses critical challenges not only to security but also to societal well-being and the population’s resilience. Indiscriminate attacks targeting unarmed civilians elicit fear, anger, and profound emotional distress, with effects that extend well beyond those directly harmed. Repeated exposure to terrorism has been associated with heightened psychological distress, increased risk of post-traumatic stress disorder (PTSD), and physiological stress responses that burden public health systems [1]. Such attacks are designed to advance a political cause by triggering fear, dread, or anger in predominantly civilian populations. For individuals, such attacks present direct physical threats to life and limb, and indeed cause fear, dread, and anger, along with emotional distress [2]. Nevertheless, not all terror attacks are equal. The expected harm, the identity or nature of the attacker, and the tactics used all matter for how the public sees a terrorist attack.

Terrorism has many faces, the latest of which is digital. From a psychological perspective, such attacks disrupt core feelings of safety and control, activating perceptions of a national threat that shape psychological reactions and policy attitudes [3]. In recent years, we have seen an increase in civilian exposure to a new form of terrorism: cyber terrorism, meaning destructive computer-based attacks targeting digital systems [4]. Cyberspace has become an indispensable part of our society, leading to a significant increase in the number of cyber attacks on critical national infrastructure aimed at harming civilians [5,6]. These attacks extend beyond digital damage. They disrupt environmental systems critical to daily life—such as transportation networks, hospitals, and utilities—and reshape the physical and psychological environments in which the threat is experienced [4]. Given that these infrastructures underpin essential services, such disruptions can have far-reaching effects on people’s health, safety, and well-being. Interruptions in hospital systems, transportation networks, and energy supplies can compromise access to urgent medical care, create public safety hazards, and heighten psychological distress. These disruptions illustrate how cyber terrorism poses challenges not only for security systems but also for the public health preparedness and mental health resilience. Crucially, the targets of cyber attacks are not always merely digital assets such as databases and management systems. Recent years have seen a growing convergence between cyber and physical systems, such that a cyber attack has the potential to immobilize a country’s or region’s electrical or railway infrastructure, disable military defense systems, and even imperil nuclear stability [3,4]. Equally, just as cyber attacks have implications for physical space, a conventional attack can disrupt critical digital systems.

Attacks carried out with the aid of computers and against them might seem less dangerous than conventional terrorist attacks, but the increasing convergence between the virtual and the real belies this assumption. Indeed, several major attacks have sought to target vulnerable critical infrastructure, with potentially life-threatening and economically devastating consequences. Notable examples include the WannaCry ransomware attack in 2017, which crippled hospital operations in the United Kingdom [7], and the MOVEit ransomware attack in 2023 [8], which compromised sensitive data globally and affected numerous organizations across various sectors. Similarly, the SolarWinds cyber attack in 2020 [9], which targeted the widely used Orion network management platform, distributed malicious code to thousands of customers, including multiple U.S. government agencies and Fortune 500 companies. Such attacks highlight vulnerabilities in critical infrastructure, including European and American nuclear, hydro, and electric systems. Beyond financial and operational harm, such attacks can destabilize healthcare delivery, restrict access to essential services, and generate population-wide uncertainty and anxiety. These cascading disruptions illustrate how digital security failures can escalate into public health challenges.

In response to the growing terror threat, whether conventional (i.e., involving traditional attack methods such as bombings and shootings) or cyber, governments have developed a range of counterterrorism policies, including the growing use of digital surveillance technologies. Such technologies, such as tools for extracting data from private online communications, or facial recognition systems, allow authorities to monitor citizens’ activities in both the electronic and physical realms, with the aim of anticipating and thwarting terrorist threats [10]. However, surveillance strategies also have significant psychological and public health implications. Research shows that heightened perceptions of constant monitoring can increase stress, undermine perceptions of control, and erode trust in institutions, all of which influence people’s resilience, health behaviors, and broader well-being [2]. Thus, while these domestic surveillance programs help countries identify potential threats and fight terrorism, they also intrude on citizens’ privacy, raising complex ethical issues. In democracies, public support for surveillance-whether physical or digital-may determine its feasibility in the long term. As such, it is crucial to understand public attitudes toward these phenomena and the tradeoffs between them.

All of this raises significant questions for political science. How does exposure to cyber terrorism affect public support for different policies? By what mechanisms does exposure to different kinds of terrorism lead to shifts in political attitudes? How do the effects of exposure to cyber terrorism differ from the effects of conventional terrorism and political violence? Do citizens feel differently about digital surveillance than about physical surveillance, and in what ways?

An emerging body of research has begun to investigate the political consequences of cyber terrorism [11], particularly counterterrorism policies such as surveillance, [9,12]. The psychological dimensions, including threat perceptions, mental health, and broader public health, remain underexplored. Cyberattacks on infrastructure such as transportation, energy, and hospitals not only pose security risks but also create conditions that can heighten people’s psychological distress, collective anxiety, and the strain on health systems. This body of work has begun to reveal how citizens respond politically to terrorist threats of different kinds, and to subsequent government policies. However, this literature is still in its infancy.

We take this research a step further, exploring the public’s attitudes toward domestic surveillance, deployed in the name of counterterrorism, following exposure to conventional and cyber terrorism. Specifically, we add to the research by examining public justifications for surveillance, as a counterterrorism measure, in response to lethal terrorist attacks, and whether these attitudes differ depending on whether the attack is carried out through conventional or electronic means. We further ask whether perceptions about national threats, defined as perceived threats to the country’s security, economy, and values, play a role in the relationship between exposure to terrorism and support for surveillance.

To investigate the conditions under which external threats may foster public support for expansive and intrusive surveillance policies, we conducted a controlled, randomized experiment using computer-generated virtual reality (VR) technologies. This relatively new method allows researchers to create situations that seem more real and authentic than more traditional experimental methods. These situations elicit emotional and cognitive responses that closely mirror real-world reactions to threats. Such immersive approaches not only strengthen the ecological validity of the research but also make it possible to examine psychological processes with relevance to mental and public health as inferred through perceived national threat and support for surveillance, in a safe and controlled environment [13,14].

We created two scenarios of lethal terrorist attacks targeting railway infrastructure in Israel. The first one involved a conventional bombing and the other a cyberattack that disrupted the railway’s electronic operational systems. By leveraging VR technology, we constructed a highly realistic yet controlled experimental environment, enabling us to study the psychological and political impact of terrorism in a safe yet immersive context. Immersive VR enables the creation of realistic environmental contexts of exposure to terrorism, allowing participants to engage with lifelike simulations of critical infrastructure disruptions while maintaining ethical control over the experimental conditions. By focusing on both conventional and cyber terrorism, our study identified differences in the responses to these forms of terrorism and their implications for security policies, through psychological processes of perceived national threat and their relevance to mental health and public resilience. Understanding how exposure to terrorism shapes perceptions about national threats and support for surveillance can inform interventions to reduce terrorism-related psychological distress, strengthen the preparedness of the public health system, and improve the population’s resilience in the face of evolving threats. This approach not only advances the literature on terrorism and security but also offers insights into how exposure to threats influences resilience, preparedness, and the evolving trade-offs between security and civil liberties in an era where cyber threats increasingly redefine political violence.

### 1.1. Political Consequences of Terrorism

Terrorism intentionally targets the democratic fabric of society. In particular, it exploits the inherent tension between the basic need for security and the desire to maintain democratic values [2,15,16]. At times of heightened terrorist activity, particularly when successful acts of terror result in severe losses, and when direct confrontation with the perpetrators of terrorism is either impossible or does not guarantee public safety, citizens’ emotional distress is frequently translated into support for radical and non-democratic counterterrorism practices. Scholars have found associations between terror attacks and changes in voting behavior [17], changes in attitudes toward members of minority groups and migrants [18,19], support for more militant paramilitary groups [20], demand for foreign and domestic counterterrorism policies, including aggressive military actions and retaliatory attacks [2,21], and support for the erosion of civil liberties [22,23]. A review of post-9/11 studies reveals that exposure to terrorism also drives support for domestic counterterrorism in the form of intrusive surveillance policies that allow governments to monitor private communications [2,24].

### 1.2. Cyber Terrorism and Digital Surveillance

Governmental surveillance is not a new phenomenon. Indeed, given the history of governments adopting and deploying surveillance powers to control their populations and suppress dissent, the notion of surveillance has incurred almost universal infamy. While this is, of course, chiefly true for authoritarian states, Western democracies have also monitored the activities and communications of their own citizens. For instance, in the United States, when domestic security became a top priority during the Cold War era, the FBI, CIA, and NSA kept tabs on social justice activists and anti-war protesters—leading many Americans to be fearful about expressing their political views, even in private communications. When the extent of the surveillance was revealed, the American Congress and government agencies enacted laws [25] and policies [26] to limit its use. Guided by the notion that law enforcement and intelligence agencies could not collect information about Americans unless there was individualized, fact-based suspicion of wrongdoing, these new laws and policies significantly constrained the government’s improper domestic spying, for the most part, without negatively affecting national security.

In the post-9/11 world, the emergence of new terror threats led to a shift back in favor of domestic surveillance. At first, these threats largely took conventional forms. For example, a succession of bombings, shootings, and vehicle-ramming attacks were carried out in central European cities [27]. However, in recent years, terror organizations have adopted cyber tools to launch increasingly sophisticated attacks [28], with cyber attacks on critical infrastructure increasing tenfold in the decade ending in 2019 [29]. Potential objects of cyber attacks include water filtration plants, oil pipelines, food manufacturing facilities, and other foundations of modern life [24]. Hospitals, with their control over sensitive patient information and the high stakes for patients’ lives, are lucrative targets for ransomware attacks [30]. The 2017 WannaCry ransomware attack in the UK exposed hospitals’ vulnerability. In 2020, a patient in a German hospital died due to a ransomware attack, underscoring the life-and-death consequences of such attacks. Similarly, a 2022 U.S. hospital attack not only compromised data but disrupted services. Public transportation systems are also at risk. The combination of interconnected systems and data-driven operations has made the railway sector, in particular, an attractive target. Attacks targeting critical infrastructures can lead to significant disruptions, including service interruptions, safety hazards, financial losses, and even loss of life and limb. Unsurprisingly, the public continues to express mounting nervousness about the destructive capacity of cyber terrorism [16].

In the U.S., under the 2001 Patriot Act and a series of other measures, counterterrorism and intelligence institutions have prioritized the development and deployment of new digital surveillance technologies to identify and monitor security threats. However, given the negative history of state surveillance in both authoritarian and democratic countries mentioned above, citizens are rightfully wary of surveillance technologies. Opinion leaders often portray surveillance as inherently infringing on citizens’ privacy and personal liberty, sometimes even as an insidious lever of control by conspiratorial elites (e.g., Al Gore: “Is it just me, or is secret blanket surveillance obscenely outrageous?” [31]; Edward Snowden: “I can’t in good conscience allow the US government to destroy privacy” [32]). At the same time, high-profile cases of intelligence agencies breaching the limits of their authority have become public knowledge, in particular following the Snowden revelations [33] and the NSO Pegasus spyware scandal [34]. These revelations have made citizens sensitive to and suspicious of government security programs, which they see as subjecting data concerning citizens’ communications, movements, and behavior to intrusive and arbitrary analysis.

All of this has aggravated the long-standing dilemma of how to balance the right to privacy with the need for security. Supporters of surveillance prioritize security over civil liberties and human rights [35] and argue that better security helps citizens feel empowered and in control. Detractors maintain that the ostensible benefits of surveillance can never justify its intrusion upon civil liberties, and that terrorism should be fought by democratic means even at the expense of security [36,37].

We note here that the public at large lacks a detailed understanding of the technical aspects of digital surveillance, or the degree to which various types, methods, and techniques infringe on personal liberty. Therefore, it is difficult to know how citizens distinguish between different objectives, types of data, and techniques of surveillance when assessing the perceived infringement of personal liberties by digital surveillance. However, lay people do appear to distinguish between the collection of personal content (geolocation, biometrics, Internet of Things, etc.) and bulk metadata collection. The literature suggests that the heightened public sensitivity to government surveillance relates to personalized surveillance policies rather than metadata [23].

The picture painted above is true generally in Western countries, and to a greater extent specifically in Israel. Israel faces the ongoing threat of terrorism. Over the years, it has deployed a variety of sophisticated technologies and tactics to monitor people’s movements and communications both domestically and at its borders [38]. In 2002, the Israeli government established the State Information Security Authority to protect critical infrastructure [39] and began the close monitoring of citizens. For example, law enforcement authorities use the Hawk-Eye tracking system and Pegasus spyware to collect personal data for surveillance without clear legal restrictions [40].

Various government-owned and private firms worldwide have developed advanced surveillance systems, including monitoring and control tools designed to detect unusual patterns of network traffic, potentially indicative of cyber threats. These technologies encompass IT systems related to internet security, email surveillance, data mining, data fusion, situational awareness, and pattern and image recognition [10].

### 1.3. Theorizing Threat Perceptions as a Mechanism

Threat perceptions refer to the conscious or unconscious assessment that something or someone is dangerous. It is a basic mental faculty by which people appraise risks posed to the life, well-being, or safety of the individual (personal threats), or to the security, economy, or values of society (national threats).

Threat perceptions are heightened in situations of prolonged conflict, where seemingly unrelated events are liable to be seen as signs of immediate or impending danger. In turn, political science has long recognized that perceived threats can motivate politically relevant behavior and attitudes [41], along with other intervening psychological variables, such as distress, stress, anxiety, and anger [42]. These behaviors and attitudes include heavy-handed counterterrorism policies and increased intransigence. Terror attacks that directly transgress norms regarding the use of violence are particularly likely to evoke a sense of threat and a desire for counterterrorism actions—even when the threat is not experienced on a personal level. For instance, Hetherington and Suhay [23] established a direct association between threat perceptions related to terrorism and support for government policies that prioritize security over civil liberties, including increased surveillance via the installation of CCTV in public spaces. Threat perceptions also drove policy preferences in the aftermath of the 9/11 terrorist attacks in New York City and subsequent attacks in Madrid (2004), London (2005), Oslo (2011), and most recently in Israel following the Hamas assault of 7 October 2023.

But how does this mechanism operate in the face of cyber terrorism? It is difficult to draw direct conclusions about reactions to cyber attacks from existing knowledge about reactions to conventional attacks for two reasons. First, despite recorded cases of attacks on critical infrastructure such as hospitals, water companies, or railway systems, there are few verified instances of cyber terror attacks having lethal consequences. Recent work on the dichotomy between lethal and non-lethal attacks has established a minimum threshold of destructiveness as a determinant of citizens’ policy preferences regarding counterterrorism [12]. By contrast, a depressing number of conventional attacks have caused mass casualties. Second, people’s response to an external threat is tightly linked with their conceptualization of the attacker. Cyber attacks can be carried out without the attacker being physically present at any time, and often with no clear way of linking the attack to a specific source [9]. Thus, cyber attacks remove a factor that in conventional terrorism plays a central role in informing the public’s response.

Empirical studies focusing on the psychological responses triggered by cyber attacks have produced mixed findings with respect to the effects of perceptions about national threats. Alongside dread and uncertainty, threat perceptions emerge in this literature as a dominant response to cyber attacks. In this vein, Snider et al. [12] found that individuals were likely to respond to cyber threats in a manner similar to conventional attacks. Such threats prompted support for harsh security measures in order to reduce perceptions of national threats, even at the expense of relinquishing civil liberties. On the other hand, Shandler et al. reported that perceptions about national threats did not mediate the exposure to cyberattacks and the public’s support for retaliation [24].

One possible reason for these conflicting findings is the way the public assesses cyber threats. Previous studies have found that people have a limited ability to accurately assess security and economic threats because they know little about cybersecurity. Other factors that make such assessments difficult are the effect of cognitive biases in calculating risks, and the distorted information from voices in the public discourse and the media [33,43,44].

Given the mixed findings in the literature, we posit that when threat perceptions are heightened, both types of lethal terrorist attacks will lead to strong support for expansive surveillance. We examine this hypothesis using a virtual reality scenario to simulate two types of lethal attacks, one carried out through conventional means (a bomb), and one carried out through electronic means (a cyber attack).

**Figure 1:**
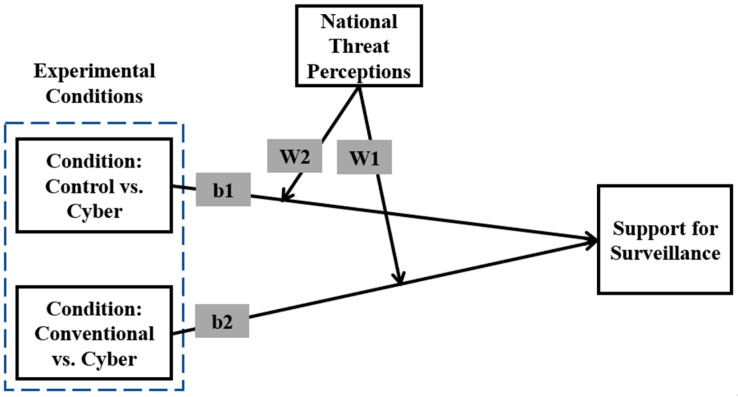
Theoretical Model.

We compare these conditions and also examine the effects of a cyberattack relative to a control condition involving no attack. Our theoretical model is presented in Figure 1. The two direct effects (b1 and b2) are moderated by threat perceptions (W1 and W2). Specifically, the model indicates that the effect of the control group compared to the cyberattack group on support for surveillance (slope b1) is conditioned by perceptions about national threats (W2). Similarly, slope b2, which represents the effect of conventional terrorism compared to cyber terrorism on support for surveillance, is moderated by W1.

### 1.4. Virtual Reality as an Experimental Tool

To test our hypothesis, we conducted a controlled, randomized experiment on 11–26 March 2021, using virtual reality to simulate exposure to two types of lethal terror attacks on the railway infrastructure. We also included a control condition using VR but with no exposure to a terror attack. In recent years, VR has emerged as a promising tool in conflict studies due to its immersive and realistic nature, which sets it apart from other media traditionally used in experiments on terrorism and political violence [13]. Among traditional methods, videos and media reports create greater emotional intensity than static stimuli such as texts and still images. They have been shown to cause shifts in psycho-political attitudes and are considered ecologically valid [9,41,42]. However, they still engage participants only to a limited extent.

In contrast to other media, VR creates a first-person experience that places participants within a fully realistic three-dimensional environment, such that they experience the (virtual) scenario directly rather than from a third-person perspective. As a result, people respond to VR experiences in similar ways as they would to equivalent situations in real life [45,46,47]. Studies using VR in research on intergroup conflict show that virtual reality simulations can evoke high levels of physiological arousal [48,49] and trigger natural behavioral responses to violent scenarios [46]. For example, individuals subjected to a virtual knife attack will not sustain physical harm, but they may experience heightened stress and anxiety, and even report feeling pain [50]. Another study found that having a (virtual) rifle pointed at oneself elicits stronger physiological arousal when experienced in immersive VR compared to viewing the same scene on a computer screen [51].

In short, VR offers an innovative approach for studying reactions to violent events in a controlled experimental setting that maintains ecological validity [50,52,53]. Moreover, VR techniques make it possible to simulate scenarios that would not be feasible to study in real-life situations, at least not in an experimentally controlled way—for practical or ethical reasons [54,55]. As such, virtual reality provides an ideal tool for conducting a controlled, randomized experiment, combining the advantages of experimental control with the realism necessary for studying real-world phenomena. This methodological approach makes VR particularly suitable for examining responses to terrorism.

## 2. Method

### 2.1. Participants

Participants were 163 Israelis, all members of Israel’s Jewish majority (the group most subject to terror attacks in Israel), and all native speakers of Hebrew. As part of our lab-in-the-field design, participants were recruited through a multi-step process designed to ensure diversity and reduce bias. Recruitment advertisements were distributed across various platforms, including social media, university forums, community bulletin boards, and public spaces outside the university campus, targeting a wide cross-section of the Jewish Israeli population. To minimize potential bias, the advertisements described the study broadly as a virtual reality research project inviting participants to take part in several immersive VR experiences. The invitation did not mention terrorism, threats or security policies, to avoid priming participants or revealing the specific research aims. Eligibility was determined through an online pre-assessment survey, which screened participants to ensure they were at least 18 years old, were native Hebrew speakers, and had no prior diagnoses of depression, PTSD, or epilepsy, mitigating potential adverse reactions to VR stimuli. No participants were excluded for epilepsy/PTSD risk.

This rigorous recruitment approach resulted in a sample balanced in gender distribution and encompassing a broad range of age groups, political ideologies, and levels of computer literacy (the latter two measures were assessed in a questionnaire completed at the end of the experiment; see under Measures below). Participants ranged in age between 19 and 71 years (mean = 30.6, SD = 9.9) and included 92 men (56.4%) and 71 women (43.6%). Participants were randomly assigned to one of three conditions: cyber terrorism (*N* = 59), conventional terrorism (*N* = 59), and the control group (*N* = 45). Descriptive statistics for the full sample and the three groups appear in Table 1.

### 2.2. Design and Procedure

For this study, we produced a 90 s professionally created, computer-generated, virtual reality simulation that vividly depicted a train derailment in Israel, followed by a news broadcast reporting the events. To test our hypothesis, we experimentally varied the framing of the derailment in the simulated news reports, such that one attributed it to a conventional attack and one to a cyber attack, depending on the experimental condition. Participants in the control condition experienced a neutral scene at the virtual train station, with the train arriving as expected, followed by a neutral news broadcast about the Israeli railway system. The experiment was approved by the Ethics Committee for Human Experiments, University of Haifa (approval 130/21).

The experiment was conducted on 7–28 March 2021. Participants were invited to our lab one by one. Upon arrival, all participants provided their written consent to be part of the study. The consent form confirmed their voluntary participation, assured them that their data would be kept anonymous, and informed them of their right to withdraw at any time. Participants were then randomly assigned to one of the three conditions (cyber terrorism, conventional terrorism, and the control condition) and underwent the VR manipulation. Following the manipulation, they completed a questionnaire containing the main measures, the covariates, and demographic information.

### 2.3. VR Simulations

During the simulation, participants found themselves in a virtual train station that resembled a real station in Israel. Using VR glasses, they could look around and observe avatars of other passengers (present in the scene as virtual characters) waiting for the next train, and a display screen indicating the train’s imminent arrival. Participants started the manipulation seated on a bench at the (virtual) train station, as if they were waiting for the train (see Appendix B for technical details of the VR setup).

When the train arrived, they were instructed to stand. At this point, participants in the two treatment conditions witnessed the train derailing, overturning, and exploding, leading to fire and smoke. The simulation included auditory cues, such as loud explosions and people screaming, to heighten the emotional impact. The virtual reality scene then faded to black, and a floating news screen with the logo of Channel N12, a well-known Israeli news channel, appeared. A caption read: “Terror attack on the Israeli railroad: Seven casualties.” For the cyber terrorism condition, the news broadcast included the lines “an unprecedented cyber terrorist attack has breached the security of Israel Railways. Terrorist hackers infiltrated the Israel Railways computer system, taking control of the operations center.” For the conventional terrorism condition, the parallel lines were “an unprecedented terrorist attack has breached the security of Israel Railways. A bomb planted by unidentified perpetrators exploded.” Full transcripts of the news broadcasts are available in Appendix A.

To ensure that any observed differences in responses could be attributed to the manipulation (cyber vs. conventional terrorism) rather than confounding variables, the two treatment conditions were designed to be as similar as possible in their sensory and emotional content throughout the VR simulation. The conditions differed only in the framing of the methods used to carry out the terrorist attack. In addition, both were described as resulting in the same number of casualties: seven fatalities, including several children, and ten critical injuries. These casualty levels were intended to ensure that the scenario met the minimum threshold of destructiveness required to affect citizens’ preferences about counterterrorism policies [21].

Participants in the control condition followed the same procedure as those in the treatment conditions, experiencing the same virtual train station environment and waiting for the train to arrive. However, unlike the experimental conditions, they did not witness a train derailment but observed the train arriving and stopping at the station without incident. They then heard a brief neutral news report (see Appendix A).

An overview of the three conditions appears in Table 2. For each condition, the left-hand screenshot shows the station during the first part of the simulation, and the right-hand screenshot is from the second part, showing the explosion/derailment in the treatment conditions and the train’s arrival in the control condition. Additional details about the VR experience, including more visuals and a description of the production process, can be found in a previous study [56] (No participants withdrew from the study due to discomfort or adverse reactions).

### 2.4. Measures

The dependent variable of interest in this study was support for surveillance, measured using two questions adapted from previous work [57]: “Are security cameras justified and effective in maintaining civilian security?” and “Should the state monitor private conversations on social networks regarding security issues?” Both questions were rated on a scale from 1 (not at all) to 6 (absolutely). Responses to the two questions were averaged to create a single score for each participant for support for surveillance.

The moderator, national threat perceptions, was assessed with a previously validated scale [5] and included four questions concerning different threats at the national level, e.g., “To what extent do terrorist attacks threaten Israel’s national security?” (α = 0.833). All questions were rated on a scale from 1 (not at all) to 6 (completely). Responses to the four questions were averaged to create a single score for threat perceptions.

Covariates included age, gender, political ideology, and computer knowledge. The original political ideology scale ranged from 1 (very right wing/very conservative) to 7 (very left wing/very liberal) and was recoded into a three-level ordinal indicator: 1 = left, 2 = center, and 3 = right. The computer knowledge scale was a composite measure assessing participants’ familiarity with computer- and internet-related concepts, including advanced search techniques, PDF management, cache storage, phishing, and Trojan malware. All demographic covariates were collected at the conclusion of the survey.

As Table 1 indicates, there were no significant differences among the experimental groups except for the mean age, which was higher among participants in the conventional terrorism group compared to the control group (F = 6.28, *p* = 002). However, this age difference was not considered meaningful for the interpretation of the study’s results. See Table A2 for correlation scores of study variables in Appendix C.

## 3. Results

### 3.1. Main Results

**Figure 2:**
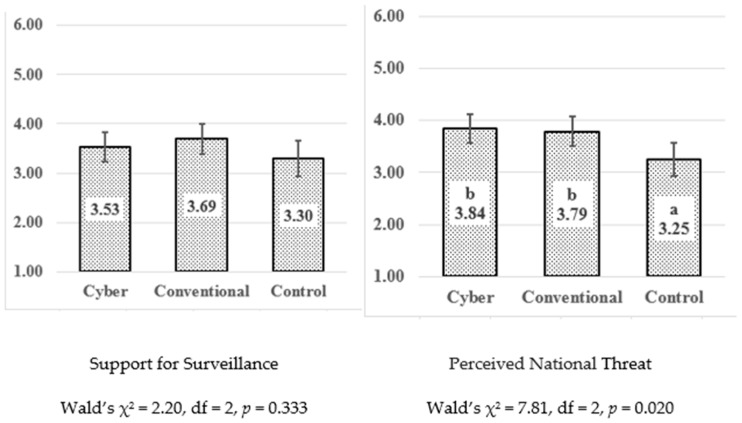
Comparison of the Main Research Measures across the Three Conditions. **Note:** Predicted marginal means; error bars for 95% confidence intervals; tests were performed including age as covariate; Latin letters for marginal mean ranking from the lowest (“a”) and upward.

As a preliminary step in our analysis, we tested for differences between the three groups with respect to the study’s main variables, the outcome policy attitudes and the perceived threat. Figure 2 illustrates the levels of support for surveillance and perceived threats in the three experimental conditions (Generalized Linear Model with robust standard error) controlled by age. The results indicate no significant effects of the condition on the mean levels of either support for surveillance or perceived threats (Wald’s χ^2^, *p* > 0.05). However, as Figure 2 indicates, there was a difference (*p* = 0.020) in perceived threats, with participants exposed to the treatment conditions (cyber or conventional terrorism) reporting slightly higher levels of perceived threats on average compared to those in the control group. (For detailed results, see Table A2 in Appendix C).

We tested the association between threat perceptions and support for surveillance. Prior to this test, an a-priory power analysis was conducted taking nine linear predictors, medium effect size (ES = 0.15) and sample size of *N* = 163, which yielded power of 98. Next, in both the control group and the cyber group, there was a very strong positive correlation between these two factors. In other words, a higher level of perceived threats was correlated with stronger support for surveillance and vice versa. Interestingly, there was no such correlation among those exposed to a conventional terror event. In that group, support for surveillance was strong, and actually stronger than the mean of the two other groups. However, it did not reach statistical significance, regardless of the perceived threat level.

Our core empirical model proposed (a) that exposure to terror attacks, whether carried out by conventional or electronic (cyber) means, affect attitudes toward government surveillance and (b) that perceptions about national threats moderate the link between exposure to terrorism and support for surveillance. To test our hypothesis, we conducted a linear regression predicting support for surveillance from the possible covariates and the three experimental conditions. Given the lack of significant differences between the cyber and control groups in support for surveillance reported above, and in order to compare the two experimental manipulations, we used the cyber condition as the reference group. We treated the control condition as the reference group in a robustness check.

In Model I, we tested for the effects of the control variables (gender, age, political ideology, and computer knowledge). Model II included the main effects of exposure to terrorism (conventional vs. cyber and control vs. cyber) and perceptions about national threats, ranked on six levels, from low to high. Model III included interaction terms between perceptions about national threats and the two experimental conditions.

Table 3 presents the unstandardized coefficients, their standard errors, and the standardized coefficients for the three models followed by 95% confidence intervals of the estimates. Model I revealed that as political ideology became more right-wing, support for surveillance rose (b = 0.28, *p* = 0.016, β = 0.190, 95%CI [0.54, 0.51]); the other control variables had no effect on support for surveillance. In Model II, only perceptions about national threats affected support for surveillance (b = 0.39, *p* < 0.000, β = 0.369, 95%CI [0.22, 0.56]), such that stronger perceptions about national threats were associated with greater support for surveillance and vice versa. This model did not show any significant main effects of the exposure to terrorism on support for surveillance. Model III captured the interaction effects between the conditions and threat perceptions. The interaction between exposure to lethal conventional terrorism (vs. lethal cyber terrorism) and threat perceptions on support for surveillance was significant (b = −0.40, *p* = 0.029, β = −0.63, 95%CI [−0.76, −0.04]). This finding indicates that the effect of exposure to lethal conventional terrorism versus lethal cyber terrorism may vary depending on the level of perceptions about national threats.

### 3.2. Estimating the Moderating Effect of Perceptions About National Threats

**Figure 3:**
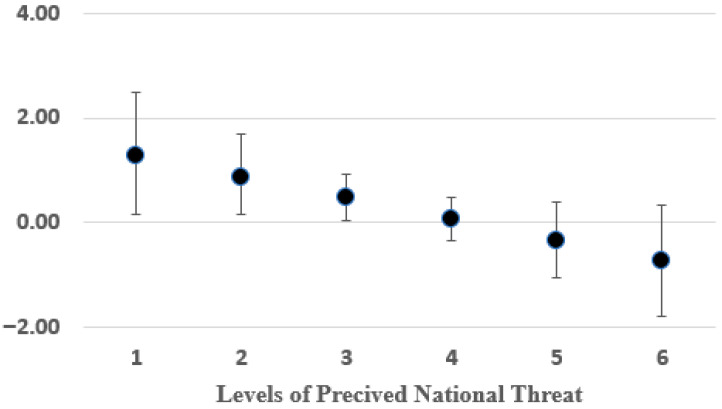
Exposure to Conventional (vs. cyber) Terrorism: Effect on Support for Surveillance by Varying Levels of Perceptions about National Threats.

To further examine the moderating effect of perceptions about national threats on the relationship between exposure to terror attacks (cyber or conventional) and support for surveillance, we utilized the PROCESS procedure (Model 1) [58] using a similar approach in Mplus [59]. This procedure enabled us to examine the sources of this interaction, as they are decomposed into separate simple slopes (see Table 4 and Figure 3).

Let *y_i_*, *ci*, *x_i_*, and *ti* be, respectively, the outcome (level of support for surveillance), the conventional exposure (versus cyber), the perceived threat level, and the interaction between the latter two (*ti* = ci*xi), for an individual i in the sample (*i* = 1, 2, 3, …, 163). Then, *b*_1_, *b*_2_, and *b*_3_ are the regression coefficients of *c*, *x*, and *t*, respectively, on *y*—i.e., marginal effects. The marginal effect of the conventional condition on the outcome is thus the partial derivation of *y* with respect to *c*, as follows:

$$\frac{\partial y}{\partial c}=b_{1}+b_{3}*x$$

The simple slopes are point estimates of the marginal effects of *c* on y for varying values of *x* (*x* = 1, 2, 3, 4, 5, 6), which represent the difference between the conventional and cyber conditions at each point of *x*.

Table 4 reports the results. The most important finding in the table is that support for surveillance differs significantly based on exposure to conventional vs. cyber terrorism only if perceptions about national threats are minimal (≤3). At higher levels of perceived threats (≥4), this difference disappears. In other words, when people sense that the level of threats to the nation is relatively low, they differentiate between types of terror attacks and decide upon the proper counterterrorism approach selectively for each type. However, when they feel that the threats to the nation are substantial, people tend to support strict counterterrorism policies across the board, regardless of the type of terror attack to which they were exposed.

### 3.3. Robustness Analysis

As a robustness check, we examined differences between participants in the control group and the two experimental groups in their support for surveillance with the control as the reference group. This approach did not result in an interactive effect (cyber: b = 0.09, *p* = 0.660; conventional: b = −0.32, *p* = 0.190). In addition, there was no significant main effect of either the conventional or cyber condition in comparison to the control group (b = 0.14, *p* = 0.550 for conventional; b = −0.02, *p* = 0.927 for cyber). However, the effect of exposure to terrorism was stronger for the conventional group than the cyber group with respect to the control group (b = 1.28, *p* = 0.031, 95% CI [0.17, 2.50]). Moreover, although the interactions were insignificant, in the cyber–control comparison, the effect of threat perceptions was stronger for the cyber group than for the control group (b = 0.57, *p* < 0.001, 95% CI [0.34, 0.80]). This was not the case for the conventional–control comparison, where there was essentially no difference between the two.

## 4. Discussion

Cyber terrorism increasingly poses risks to vital systems such as trains, water filtration plants, oil pipelines, food manufacturing facilities, and hospitals. In response to this growing threat, governments often respond by implementing enhanced surveillance measures. These policies aim to detect and prevent cyber threats targeting essential services by monitoring electronic communication networks and cyber infrastructures. However, because cyberattacks increasingly target systems that sustain daily life, such as hospitals, transportation networks, and energy supplies, their impact extends beyond digital security to the population’s health and safety. Disruptions to these infrastructures can indirectly affect mental health by heightening stress responses, perceived vulnerability, and psychological strain within affected communities Granting expansive surveillance capabilities to government agencies raises concerns about privacy rights and civil liberties, as they can lead to the intrusive monitoring of individuals’ online activities, potentially infringing on their privacy. Moreover, the collection of vast amounts of data through surveillance programs raises questions about government authority and the potential for misuse of power. As such, the effectiveness of surveillance policies in mitigating the threat of cyberterrorism remains a subject of debate. While these measures may improve the ability to detect and respond to cyber threats, challenges such as false positives, overreach, and the need for effective oversight mechanisms persist.

These developments also have significant implications for public health. Heightened threat perceptions following terrorism have been linked to greater emotional distress, anxiety, PTSD symptoms, and physiological stress responses [3,42]. These processes are relevant to understanding how perceived threat may intersect with mental health and contribute to broader patterns of public health resilience, particularly within systems already tasked with managing trauma-related outcomes. From a Conservation of Resources (COR) perspective [60] terrorism represents a profound loss of core resources, including safety, predictability, and control, which can undermine people’s mental health and strain public health systems. In this study, we pursued two primary objectives: (1) to determine whether public acceptance of surveillance, as a counterterrorism measure, varies in response to exposure to different types of lethal terrorist attacks—conventional versus cyber; and (2) to examine the role of perceptions about national threats in shaping the relationship between exposure to terrorism and support for surveillance. To address these objectives, we conducted a controlled experiment using an immersive VR platform to simulate the two kinds of terror attacks, while maintaining comparable sensory and emotional experiences. Building on recent research that identifies a minimum threshold of destructiveness as critical to shaping citizens’ counterterrorism policy preferences [12], our experimental design standardized the outcomes (seven fatalities and ten critical injuries), while varying only in the post-exposure framing of the attack as either a cyber attack or a conventional attack. This equal level of lethality was chosen to ensure that any observed differences would stem from the framing of the attack type rather than from variations in perceived severity. While this design enhances internal comparability, it should be interpreted with caution, as lethal cyberattacks remain rare. Future studies could test how varying levels of lethality shape public responses to cyber versus conventional terrorism.

By maintaining consistent outcomes across conditions, our immersive VR platform provided a controlled environment to isolate the impact of framing and threat perceptions on participants’ responses. This approach also allowed us to engage with the psychological dimensions of exposure to terrorism in a controlled, ethically responsible manner.

In terms of our first objective, we found no significant direct effect of exposure to either type of terrorist attack on support for surveillance. This outcome was surprising, as we expected that exposure to both types of attack would be associated with increased support for surveillance. We did find stronger support for surveillance in the conventional group compared with the cyber group, though not at a statistically significant level. With respect to our cyber condition, one possible explanation for this finding may be that cyber attacks are currently perceived as less immediate and emotionally charged compared to conventional terrorism, which, as suggested in prior research, often elicits stronger visceral responses [43,61]. In other words, the absence (thus far) of large-scale, lethal, real-world cyber attacks may have led participants to rely on generalized perceptions when interpreting the scenarios. This relative unfamiliarity with cyber terrorism may also have made it harder for them to fully distinguish between the two attack types. Although the distinction was embedded in the news broadcast following the VR scenario, the study did not include a manipulation check to confirm recognition, which future research might briefly add without affecting comparability.

Public awareness of surveillance tools [61] may also be limited, which could influence how participants interpret surveillance-related items. Our measure captured support for familiar, content-based indicators such as security cameras (CCTV) and monitoring of private conversations. Future work could extend this by distinguishing between metadata surveillance (e.g., tracking communication or location patterns) and content surveillance (e.g., monitoring message content) to assess whether attitudes differ across these domains.

These findings align with emerging evidence that psychological responses to cyberattacks can closely resemble those elicited by conventional political violence, particularly when the attack is presented as targeted and deadly [18,42]. This connection leads directly to our second objective, in which we posited that psychological threats are a critical mechanism underlying support for surveillance. Indeed, our findings revealed that perceived national threat significantly influenced participants’ willingness to sacrifice privacy and support surveillance measures. When they felt less threatened, exposure to conventional terrorism elicited significantly stronger support for surveillance policies compared to cyber terrorism. However, as the perceptions about national threats increased, this disparity diminished, with both types of attacks generating similar levels of support for intrusive counterterrorism policies.

These results suggest that individuals prioritize security at times when they feel very threatened, regardless of whether the threatening actors appear to be acting through conventional means or via the cyber realm. They also indicate that the effects observed in our study emerged conditionally, through perceived national threat rather than direct exposure to a specific attack type. This finding aligns with Hobfoll’s COR theory, which emphasizes that under conditions of acute or chronic resource loss, individuals mobilize behaviors aimed at regaining security—even at the expense of autonomy or privacy [3,60]. Such dynamics are psychologically adaptive in the short term but may have broader implications for population-level stress and resilience. These results corroborate prior findings showing that heightened threat perceptions amplify public support for more expansive and intrusive governmental policies [2,4,18,60]. They also suggest that lower threat perceptions enable more deliberative and balanced evaluations of social and political issues [62].

These patterns have broader public health implications, particularly for people’s mental health and psychology. Threat perceptions and emotional arousal are well-documented predictors of distress responses to terrorism. Indeed, repeated exposure to threatening imagery, even in simulated VR contexts, can promote both acute stress and long-term vulnerability. Heightened threat perceptions have been associated with emotional distress, anxiety, and even physiological stress responses, particularly in the contexts of terrorism and political violence [63].

Research on real-world exposure to terrorism [64] has demonstrated that both direct and indirect exposure to terrorism increase the risk for short- and long-term mental health problems, including PTSD symptoms, even among those not directly harmed. This work, particularly in southern Israel, highlights how continuous exposure to terrorism erodes core safety schemas and amplifies vulnerability to trauma-related distress. Our VR findings parallel these dynamics by showing how simulated exposure to terrorism—even in a controlled setting—can activate similar psychological threat processes that can have negative consequences for people’s mental health.

Recent research [65] has identified several key mechanisms linking exposure to terrorism to mental health outcomes. Specifically, having a sense of meaning in life and a lack of tolerance for uncertainty have emerged as significant pathways. Those who reported having these two characteristics exhibited better psychological well-being following the 7 October attacks of Hamas on Israel. These findings underscore how helping people create a sense of meaning in their lives and reducing uncertainty can buffer their emotional distress and promote resilience in the face of terrorism. Together, this evidence suggests that psychological responses to terrorism not only increase the risk of anxiety- and trauma-related symptoms but also shape resilience at both the individual and community levels, with downstream implications for public health policy, institutional trust, and preparedness [3].

## 5. Conclusions

Our study makes three main contributions to the literature on counterterrorism, cybersecurity, and public attitudes toward surveillance. First, it furthers the ongoing discourse on the balance between privacy and security, underscoring the central role of threat perceptions in shaping policy attitudes. While the field of cybersecurity has grown considerably in recent years, the psychological dimensions of threat perceptions remain underexplored. Our findings add nuance to this discourse by demonstrating how heightened threat perceptions, particularly following acts of terrorism, lead people to prioritize security over privacy, reflecting psychological and policy responses with mental health relevance rather than direct clinical outcomes [2].

Second, this study introduces an innovative methodological approach to terrorism research by using immersive VR to simulate exposure to terror attacks. By placing participants in vividly realistic scenarios designed to elicit physiological arousal and behavioral responses that resemble real-world events, VR allows researchers to examine psycho-political attitudes in extreme circumstances. As such, this technique enhances the ecological validity of studies assessing attitudes toward more abstract threats. For instance, simulated environments can be created to illustrate risks to critical infrastructures, such as water supplies and power grids.

Third, by incorporating a lethal cyber attack into our research design, this study contributes to the ongoing discourse within the cybersecurity community, which often hesitates to explore such scenarios due to the absence (thus far) of real-world occurrences and the significant technical challenges they present. While we acknowledge and understand these critiques, we argue that the nature of political violence is inherently evolutionary. Much of the history of terrorism is marked by perpetrators using violence in previously unimaginable ways, such as the use of passenger aircraft as weapons of mass destruction during the attacks of 11 September 2001, and the unprecedented massacres of 7 October 2023, in Israel. Although lethal cyberattacks have not yet materialized, we believe that our study offers valuable insights by preemptively identifying potential public responses to such incidents. Future research should continue to examine the possible implications of lethal cyber terror attacks compared to the current era, which is dominated by non-lethal cyber operations. Anticipating public reactions to such scenarios—particularly in terms of perceived vulnerability and emotional reactivity—can support more informed approaches to mental health preparedness and resilience planning [42].

As cyber attacks increasingly target critical infrastructure, the potential for civilian harm in the digital age becomes an urgent concern. Unlike conventional attacks, cyber disruptions to essential services—hospitals, transportation, power grids—can inflict widespread yet less immediately visible harm. Our findings suggest that the lack of large-scale lethal cyberattacks may lead to the public’s underestimation of their impact, shaping attitudes toward surveillance and security measures. This underestimation has significant policy implications. Without strong public demand, governments may struggle to justify proactive defenses, risking being underprepared for future threats.

By integrating cyber attacks into the counterterrorism discourse and leveraging immersive VR methods, our study underscores the need for a preemptive approach to mitigating civilian harm in the era of cyber conflict. Future research should also explore VR as a public health training platform, using controlled exposure to terrorism scenarios to support stress inoculation, psychological preparedness, and resilience-building interventions for both civilians and professionals such as first responders and healthcare workers. Such a proactive approach must also consider how people interpret and internalize threats, as these psychological dynamics can shape not only security policies but also the social foundations of resilience.

Overall, these findings bridge the domains of security, psychology, and public health. Understanding how exposure to cyber and conventional terrorism influences threat perceptions, mental health, and support for surveillance policies can inform strategies to strengthen the population’s resilience. This integration highlights the need for public health systems to prepare not only for the physical consequences of terrorism but also for its psychological and social impacts, particularly as cyberattacks can increasingly destabilize the infrastructures essential for daily functioning.

Taken together, this study highlights the value of immersive VR for examining real-world psychological and policy responses to terrorism, identifies perceived national threat as a central mechanism underlying these responses, and delineates the conceptual boundaries within which such findings can inform public health and security preparedness.

## Figures and Tables

**Table 1:** Background variables (frequencies, means, standard deviations) and group comparisons.

	Control Mean (SD)	Cyber Mean (SD)	Conventional Mean (SD)	Pooled Sample	Comparison Test	*P*
Computer knowledge (1–6; 6 = high)	3.89 (1.25)	4.06 (1.43)	4.13 (1.40)	4.04 (1.37)	F(2160) = 0.40	0.671
Age (Years)	27 (6.16)	30.2 (8.24)	33.71 (12.6)	30.59 (9.93)	F(2160) = 6.28	0.002
Political ideology (1 = very left wing, 7 = very right wing)	3.40 (1.16)	3.54 (1.36)	3.73 (1.48)	3.57 (1.35)	F(2160) = 0.77	0.463
Political Ideology						0.335
Left (%)	23 (51.1)	30 (50.8)	25 (42.4)	78 (47.9)	χ^2^(4) = 4.51	
Right (%)	7 (15.6)	17 (28.8)	17 (28.8)	41 (25.2)		
Gender (Female)	20 (44.4%)	27 (45.8%)	24 (40.7%)	71 (43.6%)	χ^2^(2) = 0.33	0.848
Number	45	59	59	163		

Note: Comparison tests were conducted using a one-way analysis of variance (ANOVA) and a Chi-squared test for Pearson’s test of dependency.

**Table 2:** Screenshots from the three conditions.

VR Condition	Screenshots	
**Lethal cyber attack (derailment)**	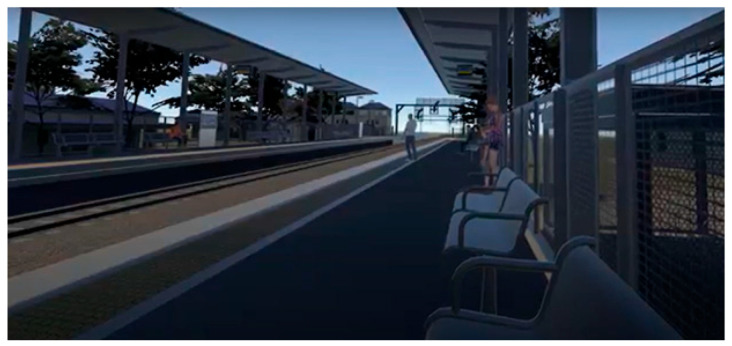	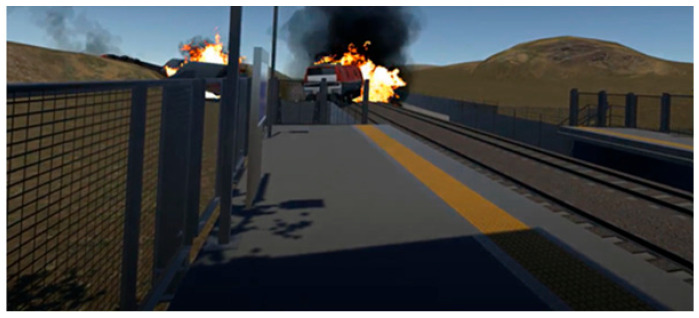
**Lethal conventional attack** **(bomb explosion onboard)**	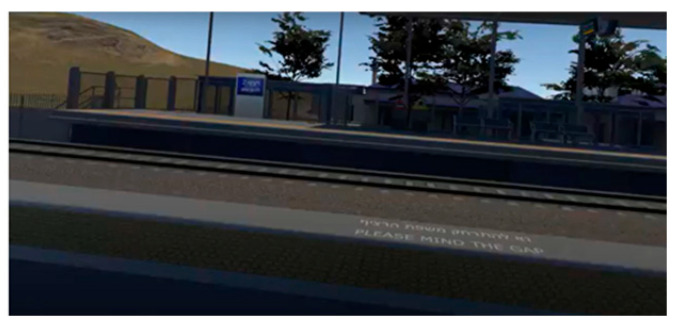	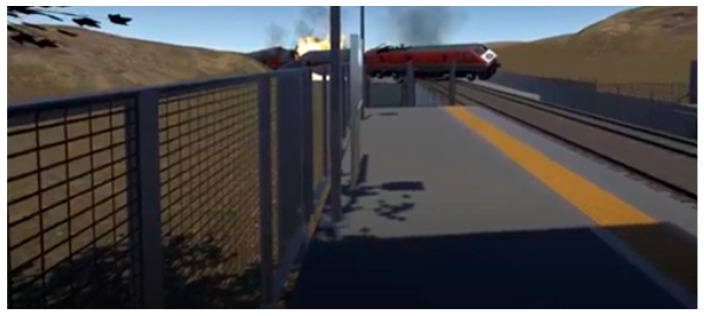
**Control** **(no attack)**	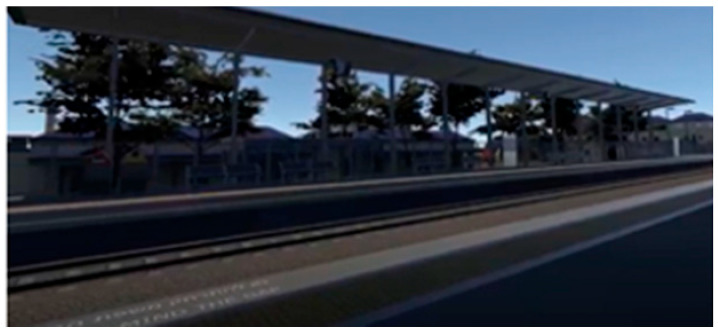	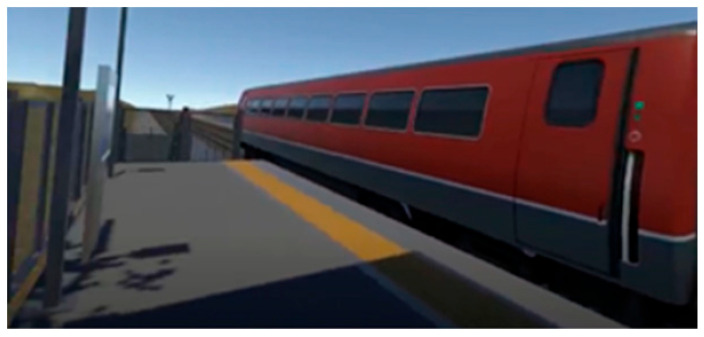

Note: The images on the left come from the first part of the respective simulation, and the images on the right are from the second part. The latter depict the derailment in the treatment conditions and the train’s arrival in the control condition.

**Table 3:** Results of Regression Predicting Support for Surveillance from Possible Covariates and Exposure to Terrorism.

	Support for Surveillance Β (Standard Errors) and β Coefficients		
	Model I	Model II	Model III
Gender (Female)	−0.10 (0.20), −0.042	−0.30 (0.19), −0.042	−0.30 (0.19), −0.124
Age (Years)	0.01 (0.01), 0.084	0.02 (0.01), 0.128	0.01 (0.01), 0.108
Political ideology (1–3)	0.28 * (0.12), 0.190	0.13 (0.11), 0.089	0.11 (0.11), 0.077
Computer skills	−0.06 (0.07), −0.07	−0.06 (0.07), −0.068	−0.05 (0.07), −0.058
Conventional vs. cyber		0.16 (0.21), 0.064	1.68 * (0.72), 0.664
Control vs. cyber		0.01 (0.23), 0.01	0.40 (0.75), 0.145
National threat (1 = low, 6 = high)		0.39 *** (0.09), 0.369	0.57 *** (0.14), 0.543
Conventional * Threat			−0.40 * (0.18), −0.628
Control * Threat			−0.09 (0.20), −0.114
Constant	3.280 *** (0.450)	1.849 ** (0.567)	1.193 (0.689)
*N*	163	163	163
R^2^	0.024	0.170 ***	0.198
Cohens *f*^2^	0.025	0.205	0.247
ΔR^2^		0.122 ***	0.028
Cohens *f*^2^		0.176	0.035

Note: The cyber condition was used as a baseline; *** *p* < 0.01, ** *p* < 0.05, * *p* < 0.1 (exact *p*-value is reported for significant effect in the text followed by 95% confidence interval). Unstandardized b for an absolute marginal effect, and standardized β for a comparative explanatory rate. Collinearity diagnosis did not reveal sources of multicollinearity in the main effect models (Tolerance > 75; VIF ≈ 1.00).

**Table 4:** Moderation Model: Simple Slopes by Level of Perceptions about National Threats.

	b (S.E.)	*P*	95%CI
Effect of exposure to conventional (vs. cyber) terrorism on support for surveillance			
Simple slopes for values of threat			
Threat = 1	1.28 * (0.59)	0.031	0.163, 2.492
Threat = 2	0.87 * (0.39)	0.026	0.151, 1.678
Threat = 3	0.47 * (230)	0.041	0.038, 0.932
Threat = 4	0.07 (0.21)	0.754	−0.356, 0.470
Threat = 5	−0.34 (0.36)	0.344	−1.047, 0.382
Threat = 6	−0.74 (0.55)	0.179	−1.806, 0.320
Effect of threat, conventional vs. cyber	0.17 (0.18)	0.357	−0.208, 0.505

Note: * *p* < 0.1.

## Data Availability

The data supporting the findings of this study will be made available by the corresponding author upon reasonable request, following acceptance of the manuscript for publication.

## Appendix A

Full Description of the Experimental Conditions and Transcripts of the News Broadcasts

After putting on the VR headset, participants were immersed in a virtual train station environment that closely resembled a real station in Israel. Participants were seated on a bench at the station and instructed to wait for the train. Using VR glasses, they could look around and observe avatars of other passengers waiting on the platform, as well as a display screen indicating that the train to Modiin Merkaz (a city in Israel) was about to arrive. After one minute, participants heard an announcement that the train was arriving.

As the train approached the station, participants in the two treatment conditions observed it suddenly derailing, overturning, and erupting into flames, with thick smoke billowing from the wreckage. The simulation included auditory cues, such as the harrowing sounds of passengers screaming, to heighten the emotional impact. The virtual reality scene then gradually faded to black, transitioning to a floating news screen prominently displaying the logo of Channel N12, a well-known Israeli news channel. Beneath the logo, a caption read: “Terror attack on the Israeli railroad: Seven casualties.” At this stage of the simulation, the two treatment conditions diverged in the framing provided by the news report.

Participants in the control condition followed the same procedure as those in the treatment conditions, experiencing the same virtual train station environment and waiting for the train to arrive. However, unlike the experimental conditions, they did not witness a train derailment but observed the train arriving and stopping at the station without incident. They then heard a brief neutral news report.

**Cyber Terrorism Condition** —The accompanying audio news report declared:

“According to initial reports, an unprecedented cyber terrorist attack has breached the security of Israel Railways. Terrorist hackers infiltrated the Israel Railways computer system, taking control of the operations center and causing the train to deviate from its course and overturn. As a result, seven passengers, including several children, were tragically killed, and dozens of others sustained injuries. At this time, all train services have been completely halted as investigations are underway.”

**Conventional Terrorism Condition**—The accompanying audio news report declared:

“According to initial reports, an unprecedented terrorist attack has breached the security of Israel Railways. A bomb planted by unidentified perpetrators exploded, causing the train to veer off course and overturn. As a result, seven passengers, including several children, were tragically killed, and dozens of others sustained injuries. At this time, all train services have been completely halted as investigations are underway.”

**Control Condition**—Participants observed the train arriving, then heard a neutral news report: “Israel Railways will purchase six railcars at a cost of approximately 60 million NIS. The Ministry of Transport and the Ministry of Finance have approved Israel Railways to execute a framework agreement with Bombardier for the acquisition of six new railcars, totaling approximately 60 million NIS. The purchase is intended to expand the railway fleet to meet growing demand in the coming years”.

## Appendix B. Virtual Reality Protocol

Two virtual environments were professionally developed using Unity software (version 2019.3) with the XR Interaction Toolkit. The experiment used an Oculus Quest 1 headset connected to a PC equipped with an NVIDIA GTX 2070 graphics card. Participants wore wireless Beats Studio 3 headphones with active noise cancellation to reduce auditory distractions. The headset display was simultaneously broadcast and recorded via Windows Game Bar for monitoring and quality control. Table A1 presents the technical specifications of the headset used in the experiment.

Participants experienced the simulation in a seated configuration to minimize movement and reduce the likelihood of motion sickness. An experimenter was present throughout each session, observing the participant’s view on the connected PC. No participants reported discomfort or sickness, and no withdrawals or exclusions occurred.

**Table A1:** Headset specifications.

Specification	Oculus Quest 1
Processor	Snapdragon 835
Display	Dual OLED, 1440 × 1600 px per eye
Refresh rate	72 Hz
Field of view	~95–100°
Motion-to-photon latency	~20 ms
Tracking latency	~20–25 ms
Audio type	Built-in spatial (open-ear)
Maximum audio output	~80–85 dB SPL
Headphone jack	Dual 3.5 mm

Audio levels were kept within a normal listening range, and no participant reported auditory discomfort.

## Appendix C

**Table A2:** Correlations Between Study Variables.

		1	2	3	4	5
1	Support for Surveillance	1				
2	Age	0.071	1			
3	Political Ideology (0 = left, 1 = center, 2 = right)	0.193 *	−0.036	1		
4	Computer knowledge (1–6; 6 = high)	−0.059	0.056	−0.05	1	
5	National threat (1 = low, 6 = high)	0.352 **	−0.156 *	0.254 **	−0.085	1

Note: ** *p* < 0.05, * *p* < 0.1.

Table A2 shows the correlations among the study variables.

**Table A3:** Measures Across Experimental Conditions.

Wald Test	Pooled Sample	Conventional	Cyber	Control	
χ^2^ = 5.49, df = 2, *p* = 0.064	3.53 (1.22)	3.69 (1.22)	3.53 (1.19)	3.3 (1.25)	Support for surveillance
χ^2^ = 5.49, df = 2, *p* = 0.064	3.63 (1.16)	3.64 (1.2)	3.85 (1.11)	3.33 (1.13)	Perceptions about national threats

Note: Standard deviations are in parentheses.

Overall, Table A3 shows no significant difference in the experimental groups in their support for surveillance. On average, participants in the two treatment groups reported slightly higher levels of perceived threats (*p* = 0.064) compared to the control group. This finding might suggest varying levels of support for surveillance depending on perceptions about national threats, indicating a potential moderating role of these perceptions.
